# Supplementary material for: Inactivation of Soybean Trypsin Inhibitor by Dielectric-Barrier Discharge Plasma and Its Safety Evaluation and Application
Source: Foods. 2022 Dec 12;11(24):4017. doi: 10.3390/foods11244017 (PMC9778619; doi:10.3390/foods11244017)
Supplement: Supplementary file 1 [file foods-11-04017-s001.zip › foods-2026043-supplementary.pdf]

Supplementary materials

Table S1. K and n values of two soymilk samples.

Sample	K	n	R^2
Control	0.0638	1.0367	0.9297
DBD	0.0627	0.8696	0.9818

Table S2. Amino acid composition and content of two soymilk samples.

Amino acid species	Content（%）	
	Control	DBD
Aspartic acid	11.70±0.3	11.66±0.0
Threonine	3.50±0.0	3.54±0.8
Serine	4.79±0.6	4.78±0.6
Glutamate	23.33±0.5	23.32±0.5
Glycine	4.16±0.7	4.18±0.0
Alanine	3.92±0.8	3.97±0.7
Valine	4.22±0.0	4.23±0.7
Methionine	1.03±0.3	0.95±0.0
Isoleucine	4.13±0.7	4.18±0.7
Leucine	7.96±0.0	8.00±0.4
Tyrosine	4.16±1.5	4.18±0.7
Phenylalanine	5.13±0.0	5.15±0.6
Lysine	6.37±0.5	6.28±0.5
Histidine	2.44±0.0	2.39±0.0
Arginine	9.86±0.6	9.82±0.6
Proline	3.41±2.7	3.37±0.9
